# Monoclonal Gammopathy of Multisystemic Significance: A Challenging Diagnosis of Light Chain Amyloidosis

**DOI:** 10.7759/cureus.72010

**Published:** 2024-10-21

**Authors:** Olasunkanmi Owolabi, Hassan O Yera, Kathryn Jenkins, Vijay Pakala, Suman Kundu

**Affiliations:** 1 Cardiology, The Shrewsbury and Telford Hospital NHS Trust, Telford, GBR; 2 Internal Medicine, The Shrewsbury and Telford Hospital NHS Trust, Telford, GBR; 3 Radiology, The Shrewsbury and Telford Hospital NHS Trust, Telford, GBR

**Keywords:** amyloidosis, biventricular assist device, cardiac mri, heart failure, left ventricle, left ventricular systolic function, light chain mgus, monoclonal gammopathy of undetermined significance, multi-disciplinary teams

## Abstract

We present a case of a 51-year-old woman diagnosed with light chain amyloidosis associated with monoclonal gammopathy of undetermined significance (MGUS). Initially, she presented with symptoms of heart failure, including palpitations, chest tightness, and shortness of breath, which were attributed to myocarditis based on cardiac magnetic resonance (CMR) imaging findings. However, her condition rapidly deteriorated, with recurrent admissions for worsening heart failure, cardiogenic shock, and stroke. A cardiac biopsy ultimately confirmed light chain amyloidosis, a rare complication of MGUS, which has a long-term risk of 0.8% in patients with light chain MGUS. Despite aggressive treatment, including chemotherapy and biventricular assist device implantation, her condition continued to decline, and she became ventilator-dependent and subsequently passed away. This case highlights the importance of considering amyloidosis in patients with MGUS and underscores the need for early diagnosis and intervention to prevent catastrophic outcomes.

## Introduction

In the United Kingdom, over 0.8 persons per 100,000 are thought to be affected by amyloidosis [1]. About 500-600 new cases are diagnosed each year, and it is the cause of death between 0.5 and one out of every 1000 people [2]. Amyloidoses are a class of protein-folding disorders characterised by the infiltration of one or more organs by proteinaceous deposits known as amyloid [3]. The deposits are derived from one of several amyloidogenic precursor proteins; the organ(s) involved and the type of amyloid will determine the prognosis of the disease [3]. Cardiac amyloidosis carries the worst prognosis of any involved organ, and light chain (AL) amyloidosis is the most serious form of the disease [3]. The importance of early diagnosis of AL cardiac amyloidosis cannot be overemphasised. If untreated, the median survival from the onset of heart failure is approximately six months [3].

Monoclonal gammopathy of undetermined significance (MGUS) affects 3.2% of adults aged >50 years [4], with light chain MGUS accounting for 1% of all cases in the <50 age group [5]. The long-term risk of developing amyloidosis in patients with MGUS is estimated to be 0.8%.

## Case presentation

A 51-year-old woman with a background of hypothyroidism, anxiety, and mild/moderate mitral regurgitation (MR) presented in August 2022 with palpitations, chest tightness, and shortness of breath.

At presentation, she was breathless and required oxygen; she had bilateral pitting pedal oedema with bibasal crepitations.

**Figure 1:**
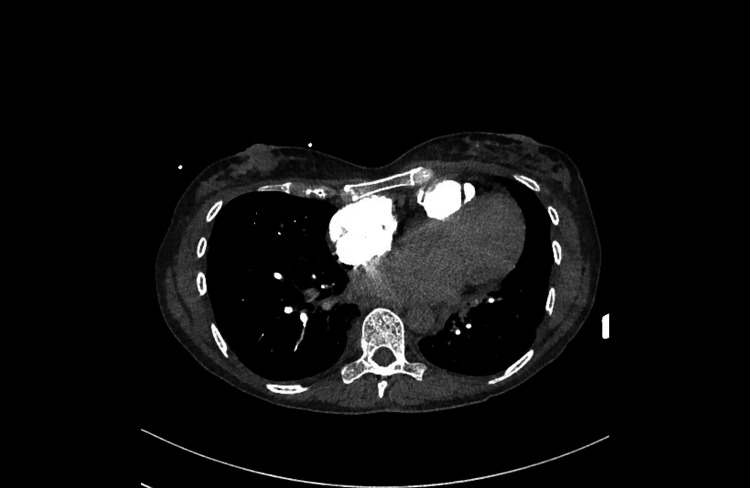
CT pulmonary angiogram showing negative for pulmonary embolism

Blood tests revealed elevated WBC of 17 × 109/L, CRP of 55 mg/L, and D-dimer of 791 ng/mL, and troponins were 94 ng and 150 ng/L. Electrocardiography showed sinus tachycardia; a chest X-ray showed cardiomegaly, pulmonary oedema, and right pleural effusion. A computerised tomography pulmonary angiogram excluded pulmonary embolism (Figure 1), and an echocardiogram revealed a non-dilated left ventricle (LV), moderately impaired LV systolic function (LVSF), moderate MR, and dilated atria with raised filling pressures (Videos 1, 2).

**Video 1:** Echocardiogram with apical 4 chamber view showing dilated atria and LV dysfunction

**Video 2:** Echocardiogram in the parasternal long-axis view

**Figure 2:**
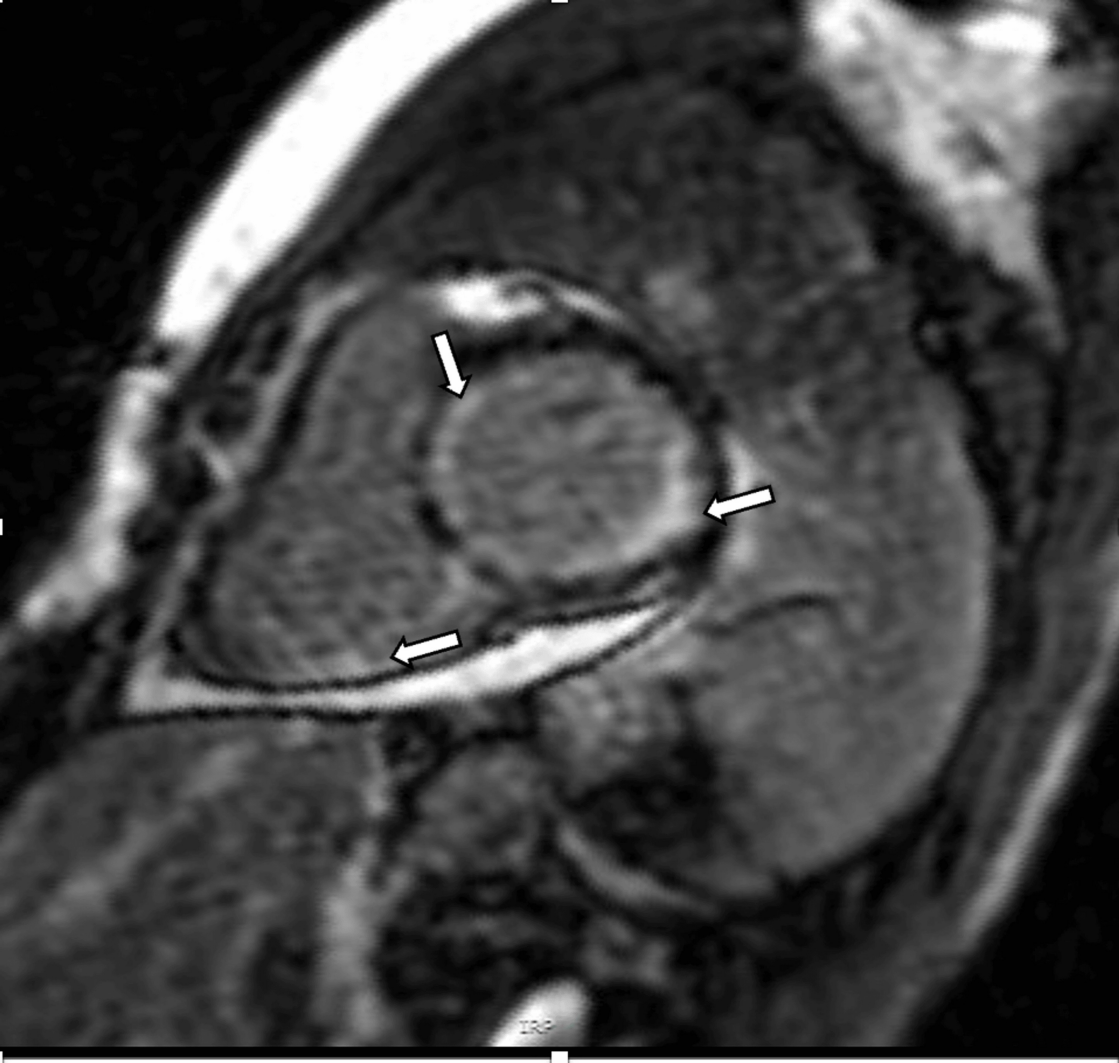
Cardiac MRI with abnormal non-ischaemic pattern LGE involving both ventricles and atria, as shown by the arrows This shows global subendocardial enhancement, the atypical pattern of LGE seen in amyloidosis. LGE, late gadolinium enhancement

**Figure 3:**
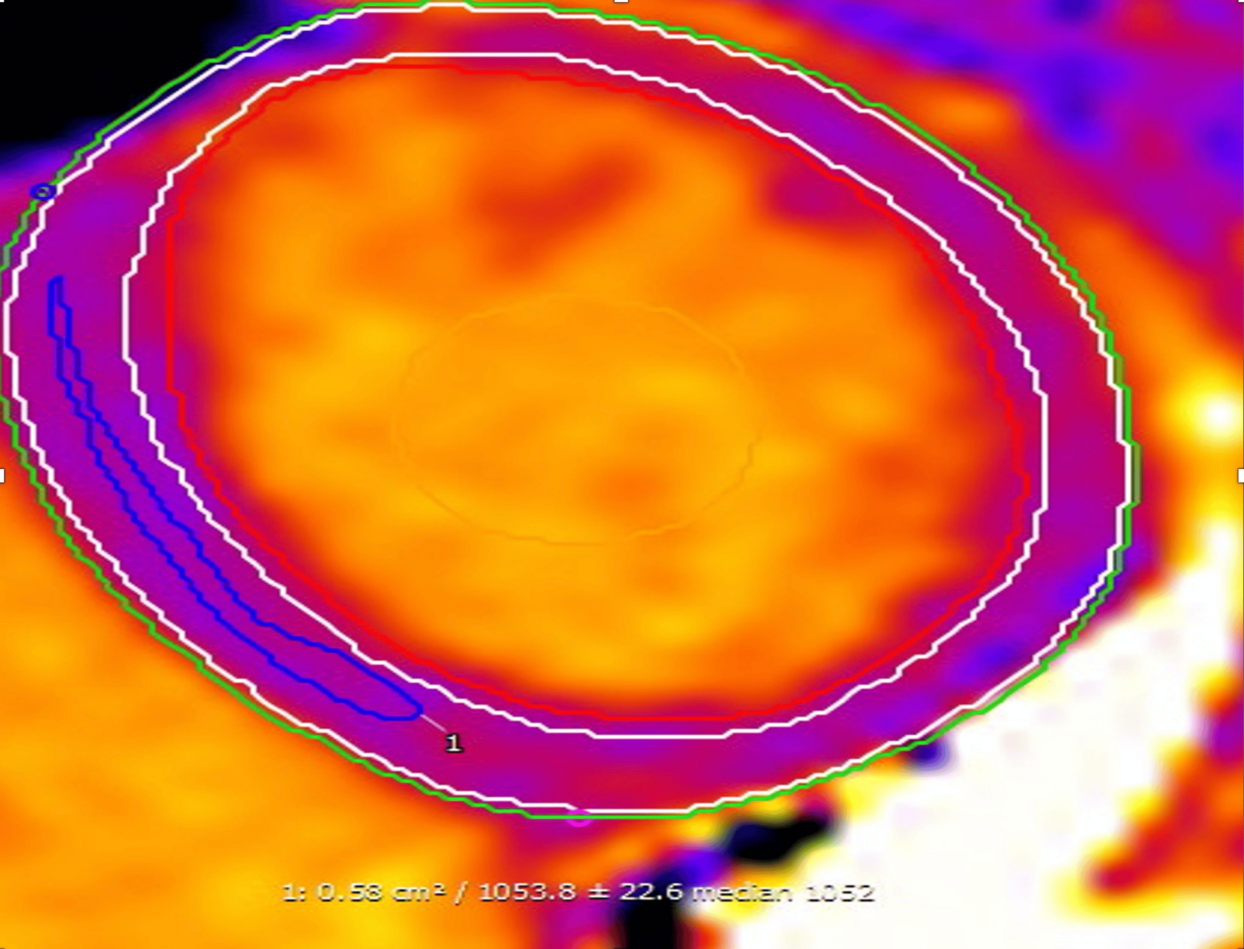
Cardiac MRI T1 map T1 map measures the longitudinal relaxation time. The normal native T1 value range is 920-1020. The above image showed a value of 1053, which is suggestive of amyloidosis.

A CMR imaging was done to determine the aetiology of the impaired LV function, which revealed a mildly impaired LVSF (ejection fraction of 46%) with an abnormal late gadolinium enhancement (LGE) pattern suggestive of amyloidosis or arrhythmogenic right ventricular cardiomyopathy (Figures 2, 3 and Video 3). However, further review of magnetic resonance imaging (MRI) images concluded that the features were those of myocarditis. She also had an invasive coronary angiogram done, which showed smooth, non-obstructed coronary arteries throughout its entire course.

**Video 3:** Cardiac MRI 4 chamber view

**Figure 4:**
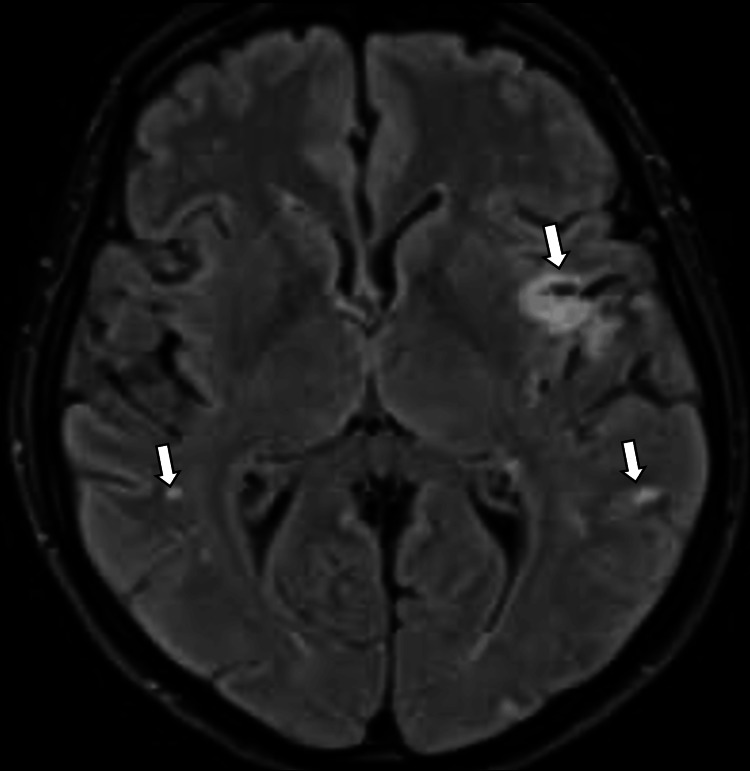
Brain MRI showing FLAIR hyperintensity and diffusion restriction within the left Sylvian cortex in keeping with acute infarct (top right arrow), small foci of infarction seen within the left occipital and parietal lobes (lower right arrow), and probable tiny infarcts within the right parietal lobe (left arrow) demonstrating multivessel territory infarcts.

She presented two weeks after the diagnosis of heart failure with symptoms of stroke. She was reviewed by the stroke team and had an initial plain computed tomography of the head, which was suggestive of an acute middle cerebral artery thrombus. She then had a brain MRI done, which showed multivessel infarcts (Figure 4). She was initially commenced on aspirin as her presentation was outside the thrombolysis and thrombectomy window. She also had a carotid Doppler ultrasound scan done as part of the workup to determine the aetiology of her stroke, which showed no significant stenosis. She was discussed at the stroke team’s multi-disciplinary team (MDT) meeting 10 days later, and the MDT outcome was a stroke with possible cardioembolic origin. This time, aspirin was switched to apixaban.

She returned two months later with worsening heart failure and cardiogenic shock, requiring inotropic support in the ICU. A repeat echocardiogram showed borderline severely impaired LV function. She recovered and was discharged back to the ward, where her heart failure medications were optimised and discharged for follow-up in the heart failure clinic.

**Figure 5:**
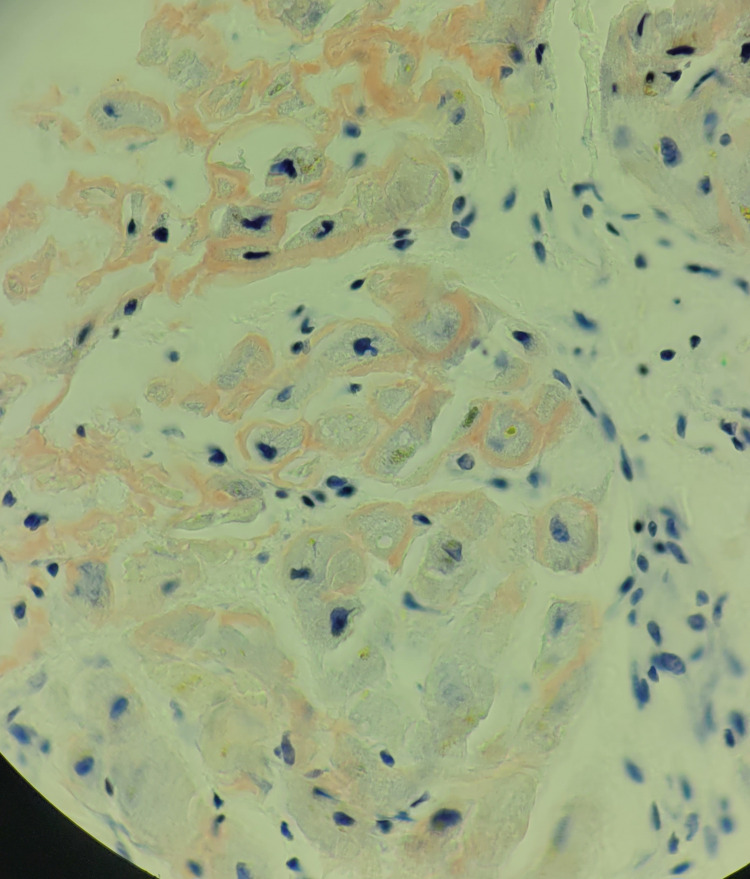
Cardia Congo red stain (100× magnification) showing birefringence under cross-polarised light

**Figure 6:**
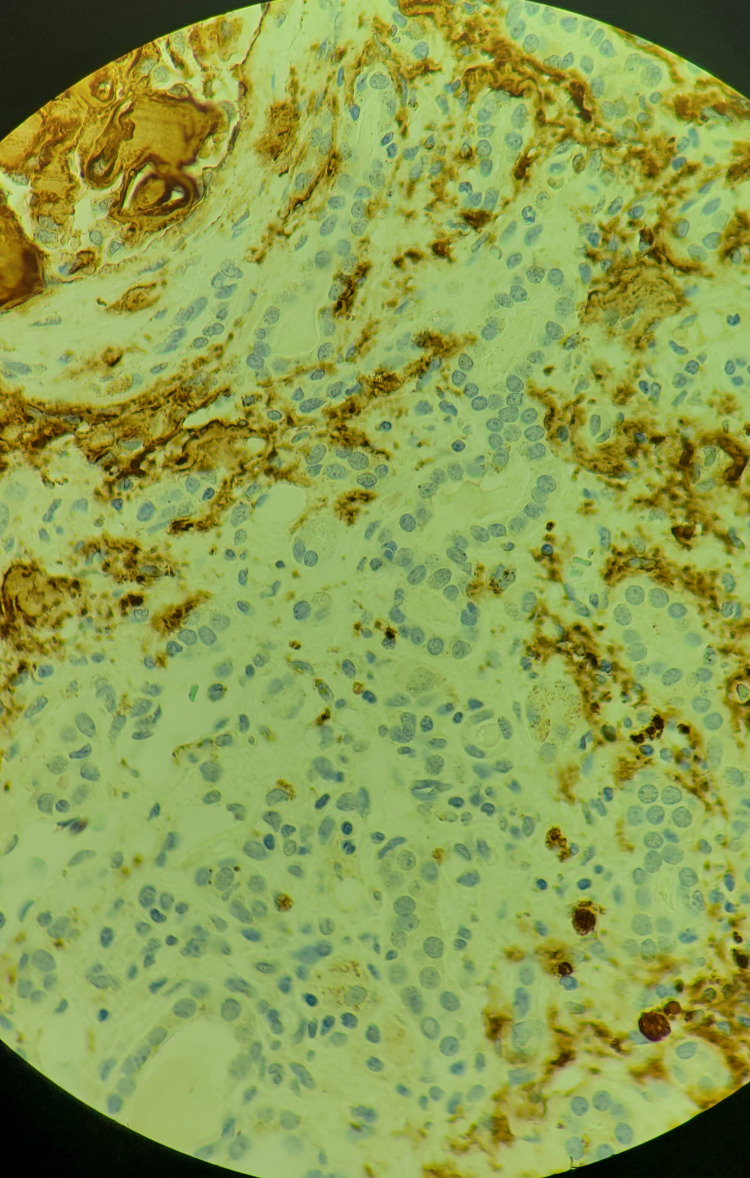
Immunohistochemical staining (100× magnification) of cardiac amyloid, which was reviewed at the National Amyloidosis Centre It stains with antibodies to lambda light chains.

**Figure 7:**
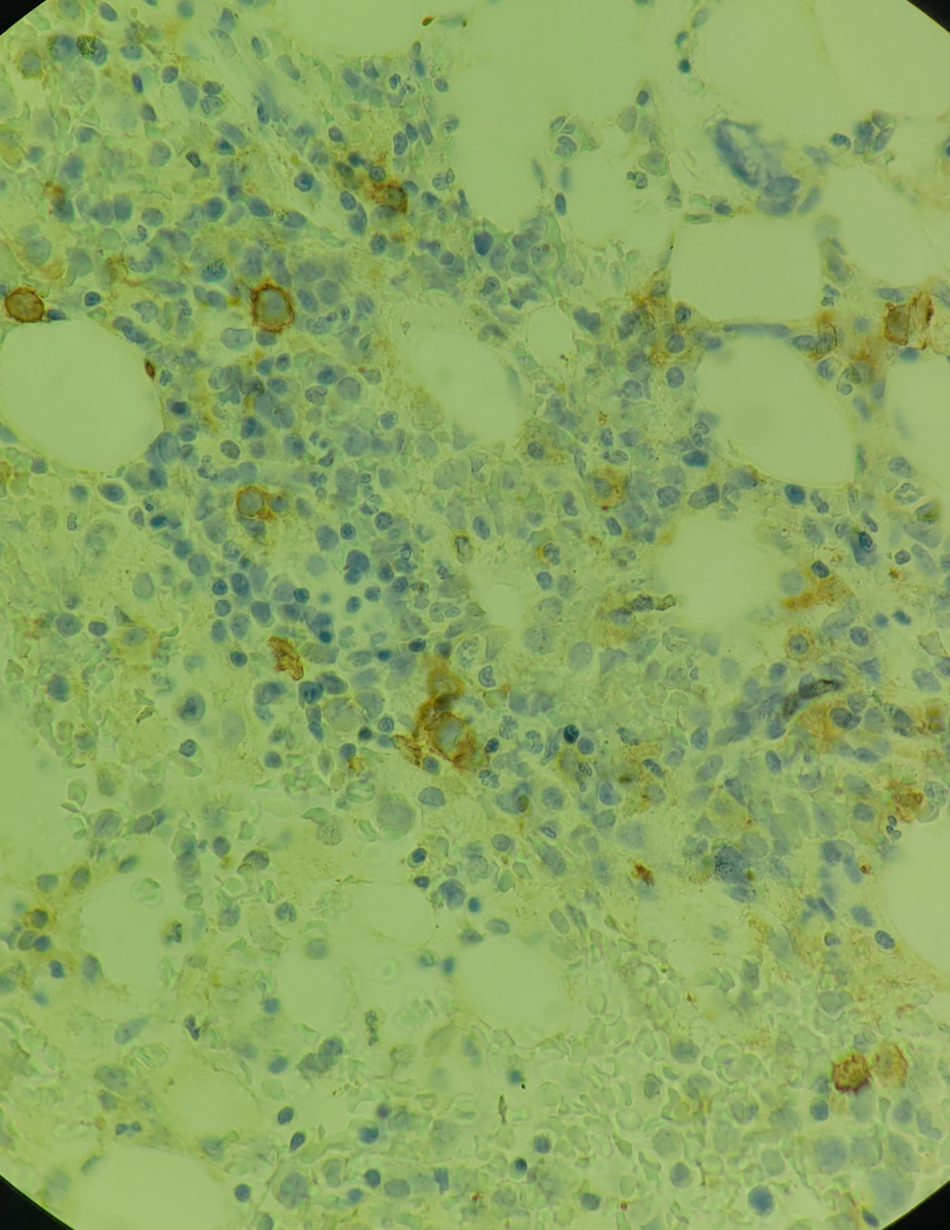
Bone marrow biopsy immunohistochemistry (100× magnification) showing a neoplastic plasmacytic infiltrate with aberrant CD 117 expression

**Figure 8:**
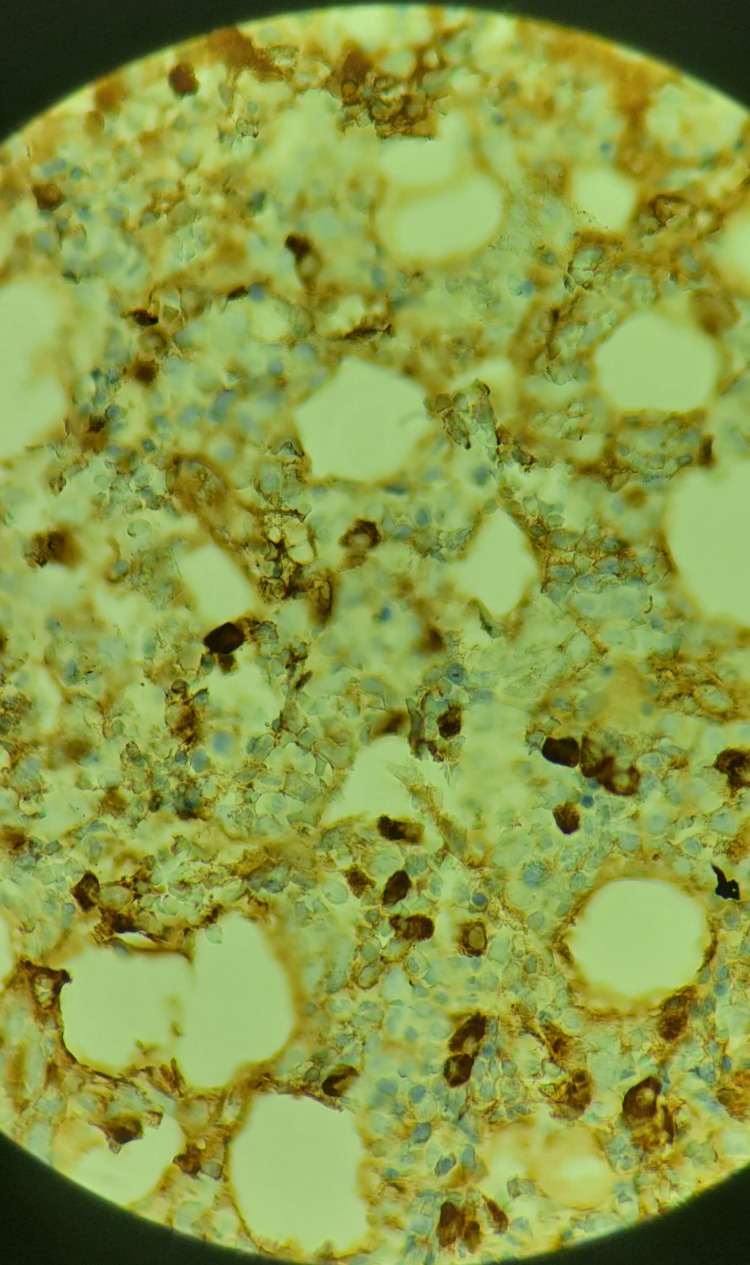
Bone marrow immunohistochemical stain (10× magnification) showing lambda light chain restricted plasma cells, which appear to constitute approximately 8% of total nucleated cells keeping with MGUS MGUS, monoclonal gammopathy of undetermined significance

In January 2023, after another admission for presyncope, she was referred to the tertiary centre for further review and care. The tertiary centre accepted her referral, but while waiting to be transferred to the tertiary centre, she was found to be in pulseless electrical activity (PEA) cardiac arrest, where she had four cycles of cardiopulmonary resuscitation before attaining a return of spontaneous circulation (ROSC). She was initially transferred to the intensive therapy unit (ITU) for inotropic support and stabilisation before she was then transferred to a tertiary centre three days later, requiring significant vasopressor support. At the tertiary centre, she had an echocardiogram, which showed severely impaired LVSF, severe MR, pulmonary hypertension, high left-sided filling pressures, and low cardiac output. Despite the optimisation of medical therapy with inotropes and vasopressors, she had no significant improvement in her end-organ function and metabolic status. She was discussed at the cardiology MDT, and she was offered a biventricular assist device to allow for the de-escalation of pharmacotherapy, aid rehabilitation, and optimise physiological status in readiness for heart transplantation. She also had a concomitant cardiac biopsy, which confirmed a diagnosis of light chain amyloidosis (Figures 5, 6), and this was followed by a bone marrow biopsy to rule out underlying myeloma as a possible cause of the amyloidosis. The bone marrow biopsy affirmed the diagnosis of MGUS (Figures 7, 8).

She was discussed with the haematology team, who commenced her chemotherapy. She could barely tolerate chemotherapy as she continued to deteriorate. She went into respiratory failure and had a tracheostomy, but she became ventilator-dependent. Due to her declining status, she was deemed unsuitable for a heart transplant, and care was focused on comfort after MDT with the family. She passed away shortly after.

## Discussion

Cardiac amyloidosis is a condition in which the extracellular space of the heart is expanded by an amorphous, fibrillar proteinaceous material known as amyloid [3]. It is important to recognise that cardiac amyloidosis is part of a systemic disease and is not an isolated condition. 

In the context of the patient being discussed, the echocardiogram primarily showed a non-dilated LV, moderate systolic impairment, moderate MR, and dilated atria with raised filling pressures. The echocardiogram features are a potential indicator of various cardiac conditions, including amyloidosis. Amyloid infiltration of the atrium can cause atrial contractile dysfunction, and thrombus formation may occur, even in this setting of sinus rhythm [3]. Thromboembolism may manifest early in the disease, and unless an attending clinician is aware of the possibility of left atrial systolic dysfunction associated with amyloidosis, the source of neurological or systemic embolism may not be recognised [3]. The patient had a multivessel stroke, which was thought to be cardioembolic in origin, although an echocardiogram did not reveal any thrombus in this case. However, it is important to note that cardiac amyloidosis can predispose to thromboembolism. Other features of amyloidosis that could be found on an echocardiogram include concentric LV thickening, atrial strain, and speckle strain of the LV [3].

The cardiac MRI presented challenges in diagnosis, initially suggesting amyloidosis or arrhythmogenic right ventricular dysplasia but later revised to myocarditis. This highlights the complexity of the diagnosis of cardiac amyloidosis, often requiring a high index of suspicion due to overlapping symptoms and imaging findings with other cardiac conditions. There are two main CMR features that could suggest the diagnosis of cardiac amyloidosis: (1) difficulty in nulling the myocardium following gadolinium injection and (2) a noncoronary, usually subendocardial pattern of delayed gadolinium enhancement both within the ventricle and in the atrium [3]. Although the MRI was later revised to myocarditis, blood tests like the serum immunoglobulin free light chain assay would have been useful in diagnosing AL amyloidosis. Other tests, like immunofixation of the serum and urine, can also be used to screen for primary amyloidosis. Serum and urine protein electrophoresis are tests that can help to screen for possible monoclonal proteins.

Ultimately, the cardiac biopsy revealed light chain amyloidosis. Patients with severe cardiac amyloidosis that is limited to the heart and in whom systemic chemotherapy is deemed to be too toxic to be tolerated because of their heart disease should be considered for cardiac transplantation, although these patients have a high mortality rate [3]. Our patient was listed for a cardiac transplant due to the severity of her symptoms and could barely tolerate chemotherapy; however, she was later deemed unsuitable due to progressive deterioration. 

Studies have shown that high-dose melphalan chemotherapy with autologous stem cell transplantation can be used in the treatment of cardiac amyloidosis and can help induce remission in some cases [3]. Excellent outcomes can be achieved with heart transplantation following careful patient selection and with aggressive control of amyloidogenic light chains in AL patients [6]. 

Sarswat et al. found that chemotherapy prior to heart transplant in cardiac amyloid patients with a high plasma light chain burden did not increase post-operative infections [6]. Pre- and post-heart transplant survival was similar in patients receiving chemotherapy to those who did not [6].

Light chain MGUS is a specific type of monoclonal gammopathy characterised by an increase in light chains (the smaller protein components of antibodies) in the serum. This condition can be a precursor to more serious disorders, including multiple myeloma and systemic amyloidosis [7].

Understanding MGUS is crucial; it is defined as an asymptomatic premalignant plasma cell disorder with specific criteria [7]:

(A) Serum M protein: less than 30 g/L (or 3 g/dL).

(B) Bone marrow plasma cells: less than 10%.

(C) Absence of related end-organ damage: this includes no signs of multiple myeloma or any other plasma cell disorders affecting organs [7,8].

(D) Absence of B cell lymphoma: confirming that there are no lymphoproliferative disorders known to produce M proteins [8].

Identifying the type of MGUS is critical for monitoring and assessing the risk of progression to more serious diseases. Light chain MGUS has been associated with systemic amyloidosis, as excess light chain can precipitate amyloid fibril formation, leading to tissue deposition and organ dysfunction [8]. The long-term risk of developing amyloidosis in patients with MGUS is estimated to be 0.8% [9].

All forms of MGUS can progress to AL amyloidosis, with lambda light chains being the predominant light chain class involved [10,11]. AL amyloidosis may develop in patients with multiple myeloma (10-15%) or may progress from MGUS [12]. Diagnosis must be confirmed by the detection of amyloid in an organ or other tissue biopsy, using Congo red or other histological staining and accurate typing [12]. 

This case exemplifies the necessity of an integrated approach to diagnosis, leveraging clinical suspicion, advanced imaging, and histopathological findings to arrive at an accurate diagnosis of cardiac amyloidosis, particularly when faced with overlapping clinical presentations and findings from various diagnostic modalities.

## Conclusions

This case underscores the importance of a comprehensive diagnostic approach in identifying light chain MGUS and its potential progression to serious conditions like cardiac amyloidosis, which requires careful consideration of clinical, imaging, and histopathological evidence. Prompt recognition and understanding of MGUS are vital for effective monitoring and risk assessment. Cardiologists also need to be aware of amyloidosis as a cause of rapidly progressive cardiomyopathy and acute heart failure.
